# Ireneusz Wierzejewski: treatment of amputees of the upper limb in Poznan during the Great War

**DOI:** 10.1007/s00264-021-05183-2

**Published:** 2021-08-16

**Authors:** Anita Magowska, Michał Owecki

**Affiliations:** Department for the History and Philosophy of Medical Sciences, Poznan University of Medical Sciences, Przybyszewskiego 37A, 60-364 Poznan, Poland

**Keywords:** Treatment of amputees, Ireneusz Wierzejewski, Upper limb

## Abstract

**Introduction:**

The Great War (1914–1918) caused a dramatic increase in the number of limbless invalids. Orthopaedics became the field of medicine that could offer the most effective help for those patients.

**Objective:**

This review article aims to present how new operations and methods in the field of orthopaedics spread to other countries during the Great War.

**Methods:**

Historical photographs of patients treated by being given hand prostheses are analysed and discussed as a case study of the transfer of orthopaedic techniques in Europe. The pictures were taken in a provincial military hospital, directed by Ireneusz Wierzejewski, the pioneer of orthopaedics in Poland.

**Results:**

The methods of preparing stumps for prostheses at Wierzejewski’s hospital followed the patterns of the time. In some cases, the prostheses were further modified to better help patients return to their former lives.

**Conclusion:**

The case of the Fortress Hospital in Poznań demonstrates that kinetic hand prostheses were also available in provincial hospitals. Modern orthopaedic procedures remain an effective treatment and a way to restore amputees to society.

## Introduction

Orthopaedics emerged as a branch of surgery at the turn of the twentieth century, the First World War (1914–1918), also referred to as the Great War, soon providing indisputable arguments for its development as hundreds of thousands of people needed functional hand prostheses. However, mechanical hands had not yet been designed and produced on a large scale. For the governments involved in the war, the challenge became a stimulus to focus on orthopaedics as a speciality of exceptional social and economic importance.

Over centuries, the disabled had been stigmatized as a social burden. In 1908, Konrad Biesalski (1868–1930), a German surgeon, perceived “a cripple as a sick person” who needs surgery and treatment. Nevertheless, in 1914–1918, limbless soldiers needed more than treatment. Their moral right was to return to the kind of life they had led before the war. Only orthopaedists could make that possible. From 1915, when the war planned by the Germans to be over quickly turned into trench warfare, the loss of the ability to fight became a central problem for the German Army. Patriotic German surgeons were involved in the design of an ideal mechanical hand. However, before the mass industrial production of artificial hands was launched, ad hoc solutions were needed locally. Far from the Western Front, surgeons in small hospitals used their experience in orthopaedics to provide amputees with prostheses patterned on the Sauerbruch arm (more on this later) or other types of original hand prosthesis produced by artisans. One of these surgeons was Ireneusz Wierzejewski (1867–1930), the pioneer of orthopaedics in Poland.

A collection of 58 photographs from 1915 to 1918, kept by Wierzejewski’s granddaughter and unpublished until now, documented his achievements in prosthetics. The photographs were taken to demonstrate the humanitarian, economic, and military dimensions of orthopaedics. They popularized the kinematic procedure and its usefulness for injured soldiers who, by virtue of functional prostheses, regained their well-being and ability to work. This article aims to analyse the content of these photographs to investigate how new orthopaedic procedures and methods spread in Europe during the Great War [1].

## Early concepts of an artificial hand

The beginnings of upper-limb prosthetics go back to antiquity; however, the idea of a voluntarily moved mechanical hand was developed in sixteenth-century Nuremberg [2]. The prosthesis took a fixed point from a corset attached to the trunk to overcome resistance. In the nineteenth century, the laws of physics were included in the mechanical therapy of human deformities and surgical procedures. Performing amputation of the hand with direct reference to the adaptation of a substitute was especially important, because it determined the patient’s ability to work in the future. The surgeon focused on the future adaptation of the stump to an artificial limb, rather than on aesthetics. In 1818, the first non-cosmetic hand prosthesis was constructed by Peter Bailiff, a German dentist, who was the first to utilize the residual activity of the stump. In 1845, a prosthesis made for the celebrated vocalist M. Rogers, who lost his right upper limb above the elbow, was of great interest in Europe. A Prussian mechanic, van Petersen, constructed a three-piece prosthesis, which imitated the wrist, the forearm, and the rest of the arm. It ensured free motion of the shoulder joint. However, this artificial hand was only for amputees with a stump of sufficient length [3, 4].

The theory behind cineplastic operations, introduced in 1898 by Giuliano Vanghetti (1861–1940), an Italian physician acquainted with neuroplasticity theory and other achievements of physiology, became a milestone in the development of prosthetics. Touched by the tragedy of Italian soldiers mutilated by the Abyssinians, Vanghetti wrote a series of articles on his concept of a hand prosthesis powered by the remaining muscles of the stump and stimulated directly from the brain. According to him, under the influence of physical exercises, a newly formed muscle element reaches its innervation by the mechanism of peripheral neural plasticity. Vanghetti suggested that a surgeon should be able to select tendons and muscles based on their force and function and suture them to form a tendonous-muscular loop enveloped by skin flaps. A padded ring could then be put around the stump above the radius and ulna. The flexors and extensors pulling the stump upwards would give power to the prosthesis, and the patient would be able to voluntarily bend the fingers of the mechanical hand using cords linked to this ring [5]. Not being a surgeon, Vanghetti could not verify the concept in practice. This was done in 1900 by Prof. Antonio Ceci, who performed the first cineplastic operation on a human being. After 1 month of exercises, the loop could be used to lift objects weighing as much as 12 pounds [6]. Vanghetti’s pioneering theory has never lost value and has influenced the development of modern bionic prostheses. Overcoming the limitations of nature by extending or supplementing the deformed human body with prostheses has become the mission of orthopaedics.

## Upper-limb prostheses during the Great War

The demand for prostheses grew substantially during the Great War, as mines, dum-dum missiles, and grenades devastated the human resources of all armies. The number of limbless soldiers increased to unprecedented levels, and their return to the Front depended on functional mechanical hands, which had not yet been invented. In 1915, independently of Vanghetti, Ferdinand Sauerbruch (1875–1951) described an innovative operation in which the residual muscles of the stump were stitched to form a loop with a pedunculated flap of skin to be drawn through the tunnel of the muscle [7]. The Sauerbruch arm, as it came to be known, was moved voluntarily, which meant that thousands of limbless German veterans could hold a gun and return to the battlefield. Unexpectedly, other surgeons, Erwin Payr (1871–1946) in particular, accused him of plagiarizing Vanghetti’s brain-driven prosthesis concept. Sauerbruch stated that he did not know about Vanghetti’s publications, but pointed out that the two procedures differed from each other, whereas Vanghetti used the distal part of the stump, Sauerbruch prepared the cineplastic muscle tunnel in its proximal part. The latter’s prosthesis was held on the stump by straps above the elbow and a natural leather sleeve. The only similarity was in the use of the remaining muscles of the stump [8]. However, Vanghetti’s priority was an obstacle for which Sauerbruch did not receive the Nobel Prize in 1919 [9].

In a military hospital in Singen, Germany, Sauerbruch improved skin tunnel formation in the proximal part of the stump to increase the productivity of the remaining muscles. Many surgeons tried to perform this operation, but the results were poor because they were not acquainted with plastic surgery techniques. Thus, the German Ministry of War recommended that Sauerbruch’s cineplastic operation be done only by surgeons trained in Singen. Sauerbruch obtained exclusivity in the training of kinematic operations in Germany. Working out the cineplastic procedure was not easy, but starting anew technology involving a voluntarily moved prosthesis was much more complicated and expensive. It was only in 1918, with the support of Badischer Heimatdankes from Karlsruhe and the arms manufacturer Alfons Mauser from Cologne, that Sauerbruch was able to establish a workshop in Singen. In the following year, a new distribution system for standardized upper-limb prostheses and their components was created throughout Germany. Nonetheless, Sauerbruch’s achievements soon failed because inflammation and infection were frequent in the tunnel of the stump. At the end of 1918, the procedure was modified by Max Lebsche (1886–1957), who worked closely with Sauerbruch [10].

Germany demonstrated a pragmatic attitude towards limbless veterans. From 1915, a prosthesis testing centre produced interchangeable parts for prostheses for fourteen different professions. In the early stages of the Great War, the Germans accepted the American Carnes artificial arm with a tie-rod. However, when, in 1917, the US Forces entered the theatre of war, the import of this prosthesis was suspended. German orthopaedists stressed that mechanical hands for German soldiers should be manufactured only in Germany. Otherwise, German soldiers injured by American grenades would be provided with American prostheses.

Patients who lost both arms were not given two Sauerbruch prostheses, but an attempt was made to form Krukenberg plastics on the stump. Hermann Krukenberg (1863–1935) invented an amputation procedure in which the stump is a prosthesis. He transformed the forearm stump into a pincer with two branches covered with skin and retaining the sense of touch. After training, a pincer could work as a hand. The Krukenberg operation was also recommended for the blind [11]. In developing countries, its functional results were excellent [12].

Jakob Hüfner (1874–1968) ends this list of inventors of upper-limb prostheses at the time of the Great War. He developed a mechanical hand that could be actively opened and closed [13]. The demand for prostheses has strengthened the interdisciplinary nature of orthopaedics as a speciality based on surgery, neurology, physiology, physics, technology, and mathematics.

## The first Polish orthopaedic hospital and its founder

During the Great War, the production of artificial hands in Germany was insufficient and their shortage was severe in peripheral hospitals. One of these was the 13th Fortress Hospital in Poznan (from the end of the eighteenth century, this town and other western territories of Poland had been occupied by the Germans), which was operating in 1915–1918. It had been opened in 1913 as an orthopaedic hospital for children and was organized and managed by Ireneusz Wierzejewski.

Born to a forest inspector’s family near Poznan, Wierzejewski was too poor to study at university. After graduating from high school, he took a job in an iron tools and agricultural machines factory. Working successively as a locksmith, turner, founder, and blacksmith, he acquired the skills necessary to design and construct prostheses. By 1903, Wierzejewski had saved enough money to start medical studies at the university in Greifswald, and then in Berlin, Wurzburg, and Munich, where, in 1908, he received a diploma. He took an assistant position in the Munich Clinic of Surgery and Orthopaedics, headed by Fritz Lange, who greatly appreciated Wierzejewski’s predisposition to orthopaedics. Lange could not, however, hire him fulltime, so he recommended the young physician to Konrad Biesalski, the founder and director of the newly created Berlin-Brandenburg Therapeutic and Educational Institution for the Crippled (Berlin-Brandenburgische Krȕppel-Heil und Erziehungsanstalt). Biesalski was the first to perceive someone with a disability as a sick person to be treated. In his institution, children with scoliosis and other deformities and disabilities were treated with orthopaedic appliances; others, with congenital defects and bone tuberculosis, were operated on and provided with prostheses manufactured in nearby orthopaedic workshops. Under Biesalski [14], Wierzejewski learned orthopaedic procedures and the principles of prosthesis design.

In 1910, Wierzejewski obtained a doctorate in Leipzig. In the following year, he returned to Poznan to organize the first Polish orthopaedic hospital for children with acquired disabilities. In 1913, the hospital was opened with 36 beds. Wierzejewski took the position as its director and chief physician. He also organized orthopaedic workshops [15].

In 1915, the Germans transformed the Poznan orthopaedic hospital into the 13th Fortress Hospital. Conscripted into the German Army, Wierzejewski became its orthopaedics consultant and the hospital commander. Until the end of the war, he performed surgeries and treated around 8000 wounded soldiers, primarily privates of Polish origin. Most of them needed artificial hands to return to the Army or, in line with German regulations at the time, take a job in agriculture or industry [16].

When Poland regained independence in 1918, Wierzejewski organized the Polish sanitary troops and commanded them, fighting Germany to stabilize the western state borders. Then the Fortress Hospital was closed. In 1921, the orthopaedic hospital for children was reopened and became the site of the first orthopaedic clinic in Poland, run by Wierzejewski as an associate professor. His research interests then focused on the surgical and other treatment of scoliosis, hummock, and clubfoot. In 1928, he founded the Polish Orthopaedic Society. In 1930, he died from injuries sustained in a car accident. His orthopaedic activity in the wartime was the combination of theories regarding artificial hands, knowledge of neurology, and his many surgical and other technical skills [17].

## Wierzejewski’s prostheses

**Fig. 1:**
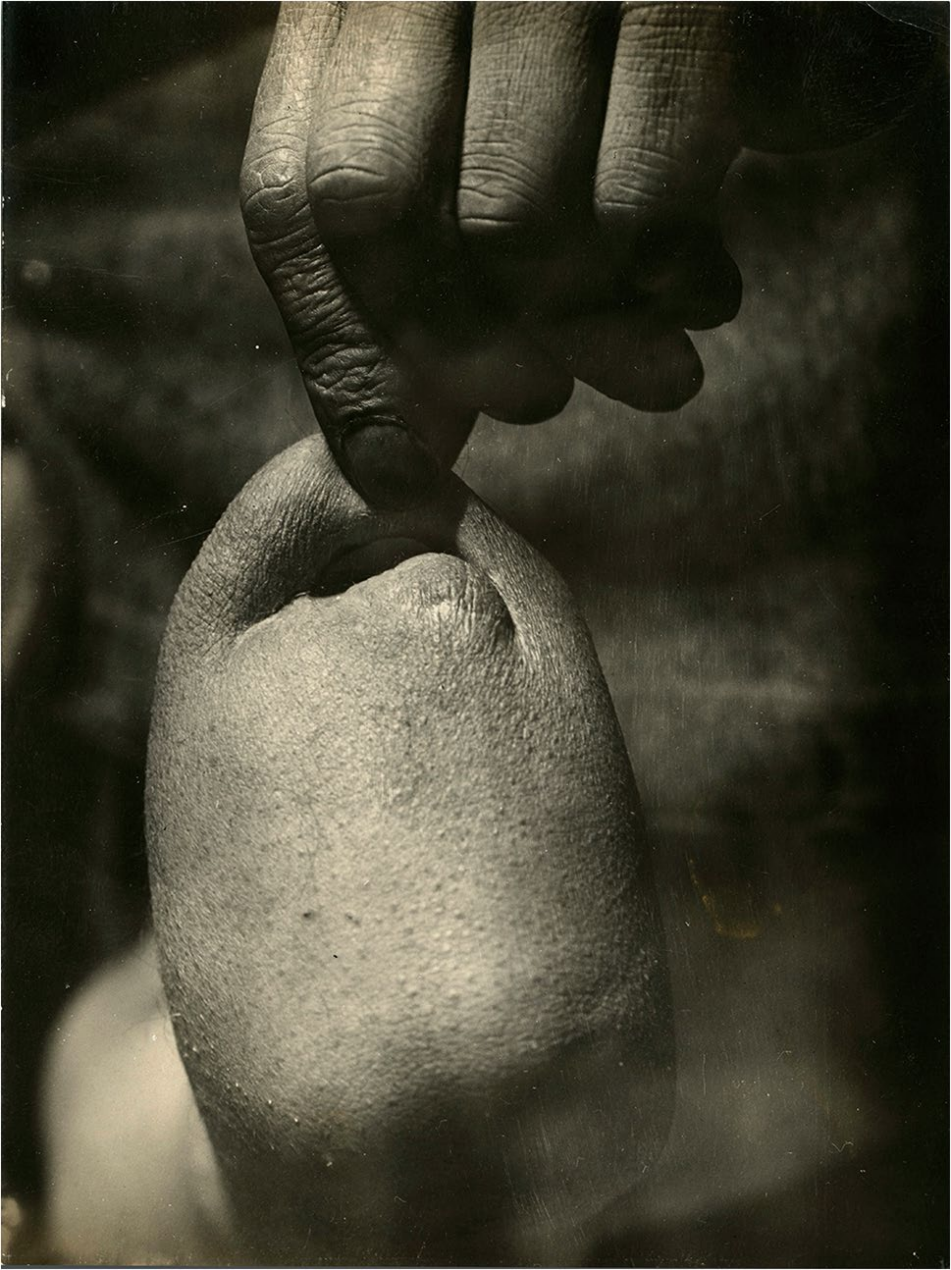
Results of cineplastic operations carried out by Wierzejewski

**Fig. 2:**
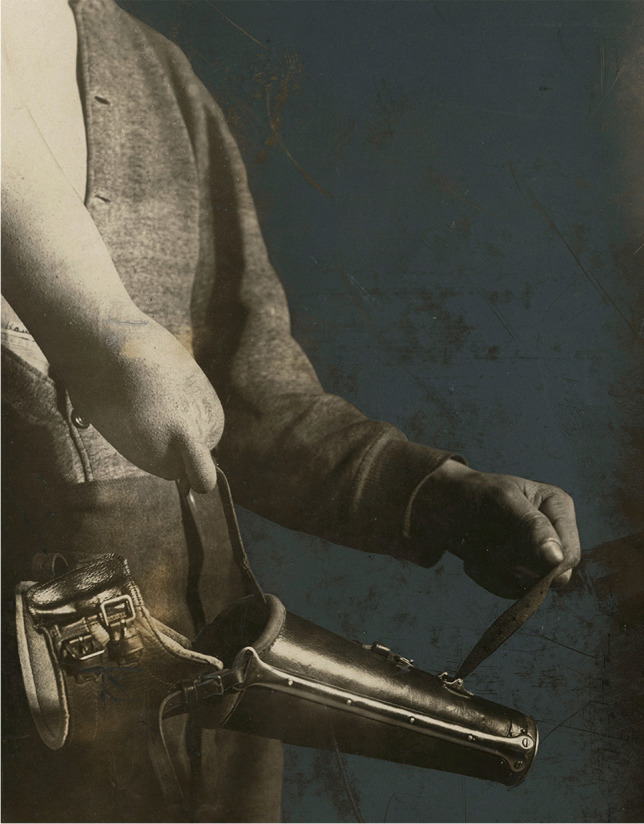
Kinetic hand prosthesis provided by Wierzejewski

Most of the patients admitted to the 13th Fortress Hospital suffered from combat injuries and bullet wounds that had damaged the peripheral nerves. They were treated with surgery, helped to regain their strength by performing gymnastics, and finally provided with prostheses if they were needed [18]. The hospital received Sauerbruch’s artificial hands in insufficient quantity. To solve the problem, Wierzejewski himself designed hand prostheses and supervised their construction in nearby workshops. His prostheses were in line with the latest standards; they were an extension of the disabled body, not simply a tool. Wierzejewski learned the cineplastic procedure from German medical journals without any practical training in this field, but he performed it successfully [19]. Stump plastic surgery became a common procedure in his hospital. His drawings of the stages of forming a tendinous-skin loop in the distal section of the stump confirm his technical skills and his abilities to independently modify patterns described in the literature. Wierzejewski used various surgical methods, each time adjusting the selection of the procedure to the patient’s clinical situation. However, the principle of using the residual stump muscle remained unchanged. He performed Sauerbruch’s procedure several times (Figs. 1 and 2).

**Fig. 3:**
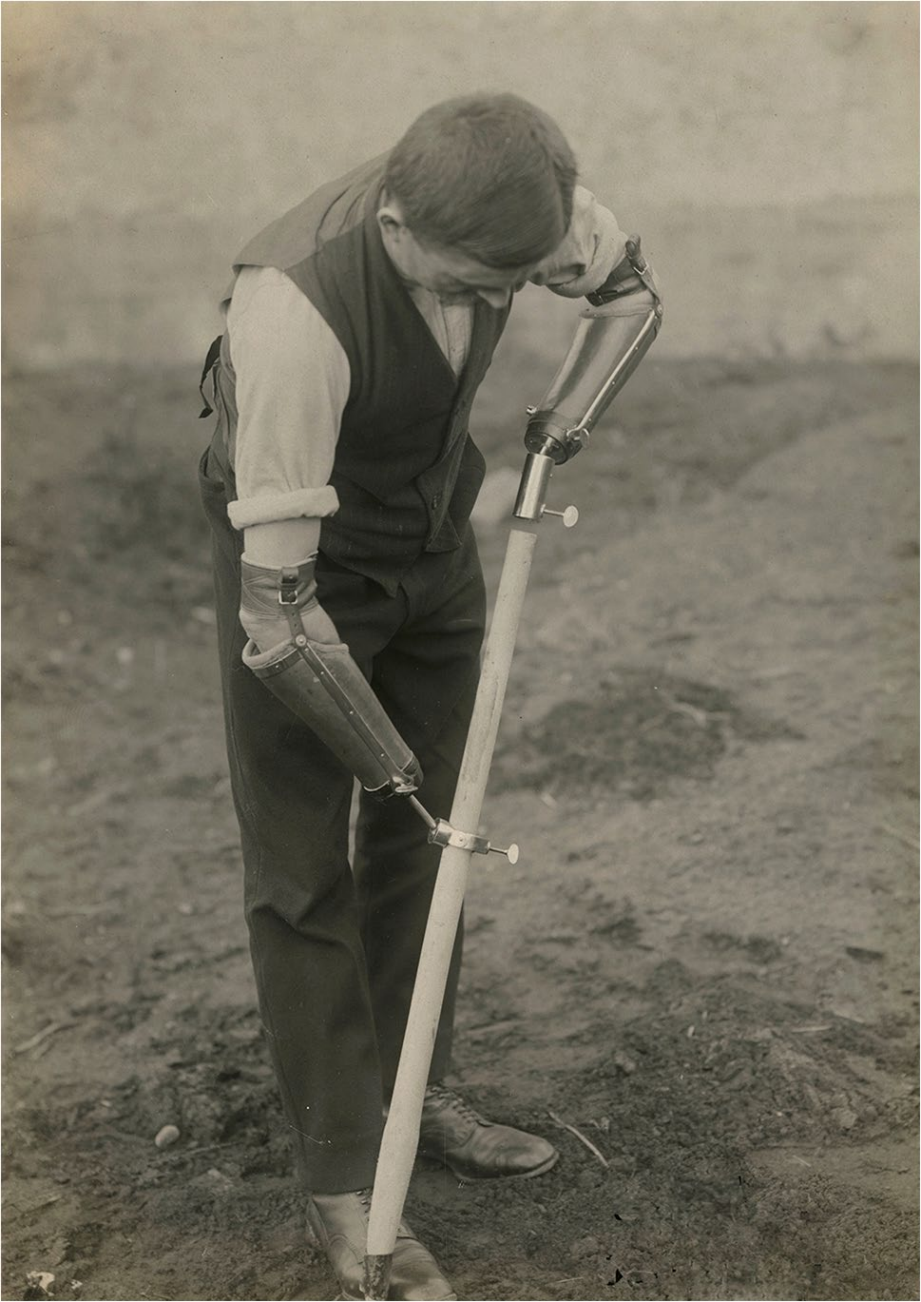
A double amputee with upper-limb prostheses

Some photographs suggest that Wierzejewski also performed Vanghetti’s plastics on stumps. It is possible that some limbless veterans lost their hands directly on the battlefield and Wierzejewski only provided prostheses manufactured in the nearby workshops for them (Fig. 3).

**Fig. 4:**
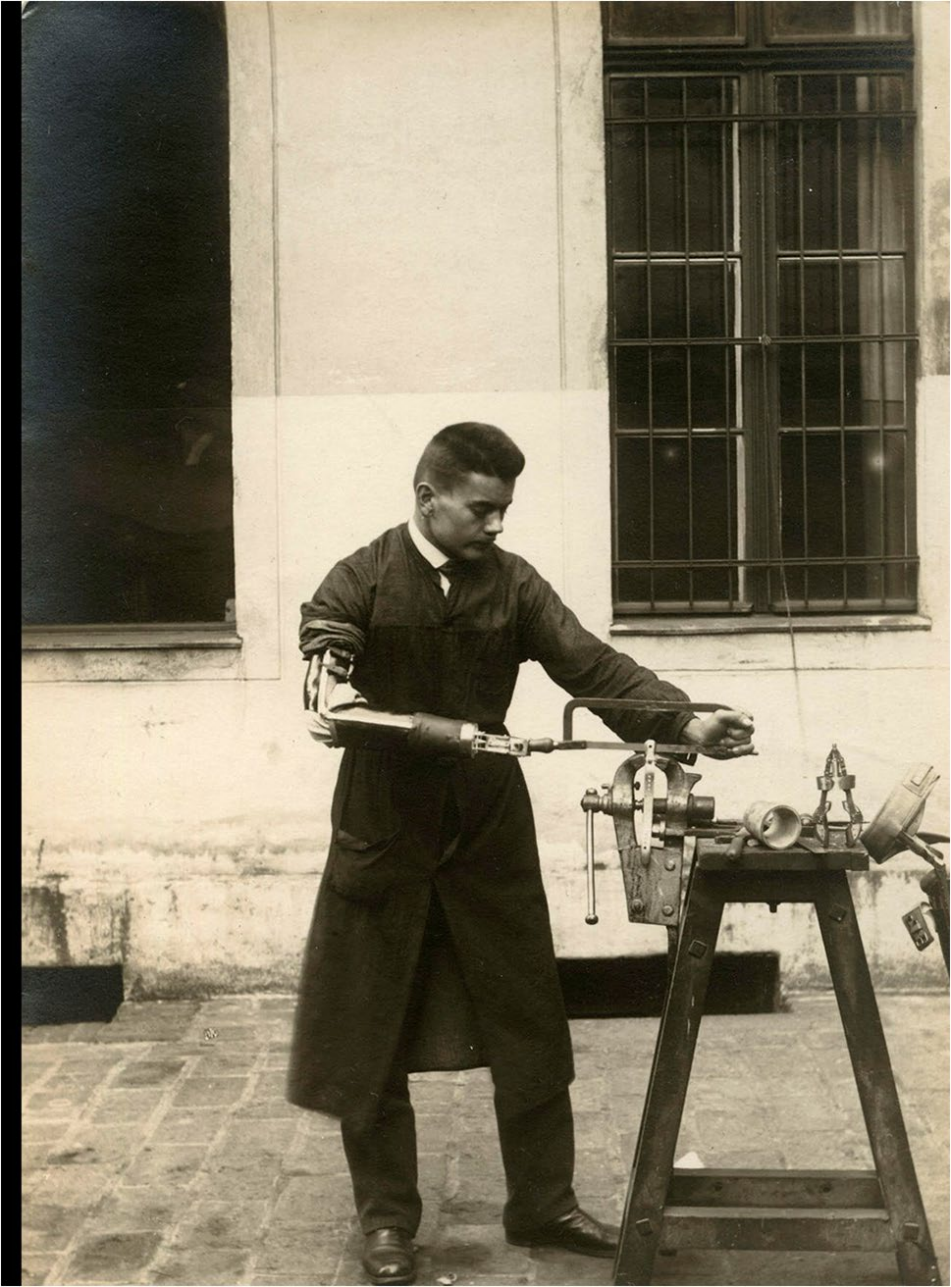
Presentation of hand prosthesis with interchangeable tips

Trained practically in metalworking and mechanics, and acquainted with advances in neurology and surgery, Wierzejewski was an ingenious designer of inexpensive and functional artificial hands. His prostheses resembled neither the Carnes arm nor the Sauerbruch hand, even with Lebsche’s modifications. Wierzejewski did not use a harness or straps to fix a mechanical hand. His prostheses were directly connected to the loop at the end of the stump and made of the materials available. The prostheses also had interchangeable tips, such as a hammer, pincers, or gripper, making labour in a factory, or craft workshop possible. They had a disadvantage, however, because they substituted the lost limb to a limited extent, enabling only such manual activities as grabbing and dropping objects, sweeping, or metalworking (Fig. 4).

**Fig. 5:**
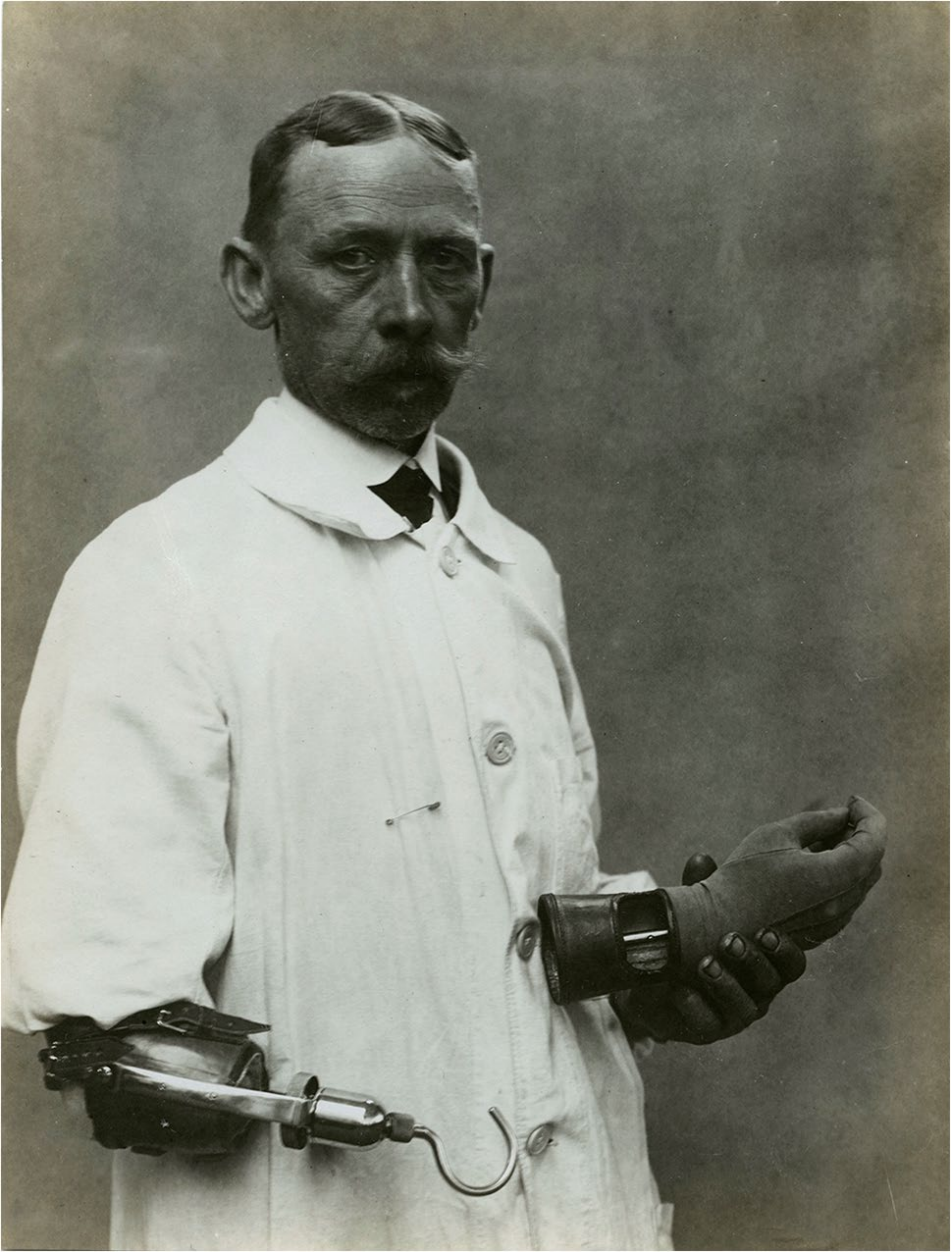
Presentation of hook prosthesis functionality and aesthetics

The hook prosthesis was cheaper but also useful. The leather sleeve camouflaged the hook and other metal parts of the prosthesis to fulfil cosmetic standards, as in the Sauerbruch arm (Fig. 5).

Wierzejewski could also improve partially fingerless hands. Skilled in the transplantation of tendon and bone, he relocated one of the patient’s toes or thumbs to restore the ability of the palm to grasp. He masked the stumps of the fingers with a leather cover or a glove.

## Conclusion

The idea of using residual stump muscles to operate an artificial prosthetic hand became technically achievable and possible to implement in surgical practice at the beginning of the twentieth century. Wierzejewski’s professional activities demonstrated that even orthopaedists from provincial hospitals at that time had a wide range of opportunities to offer this modern assistance to their patients. His achievements in prosthetics fit with the history of the Great War. He provided artificial hands constructed using the patterns of Sauerbruch’s, Lebsche’s, and Vanghetti’s prostheses. The photographs taken in Wierzejewski’s hospital and now presented above for the first time to the public also demonstrate another significant phenomenon: the potential of orthopaedics to change human minds. Bringing amputees back to society was not only a medical issue. It also needed a sensitive and empathic society. Since the very beginning of orthopaedics, its philosophical frames and mission were to give people with disabilities a better life and a dignified place in society and to restore, at least in part, lost, or disordered function of the musculoskeletal system. The photographs also demonstrate that orthopaedic technologies could change the course of history, in global and undoubtedly individual terms. Such artificial aids influenced the lives of badly injured patients invaluably—men of working-age. They restored hope to people from the marginalized fringes of society: they allowed those men to perform simple tasks, improved the self-service capacity in everyday life, and masked the frustrating stigma of disability. Kinetic hand prostheses also had a propaganda overtone, reducing the fear of recruits of a different nationality serving in the German Army. Furthermore, Wierzejewski’s inventions were a kind of business card for him, presenting his versatile talents.

## Data Availability

No applicable.

## Code availability

No applicable.
